# A case of superior vena cava reentrant atrial tachycardia after pulmonary vein isolation with pentaspline pulsed field ablation

**DOI:** 10.1016/j.hrcr.2026.03.016

**Published:** 2026-03-24

**Authors:** Daisuke Baba, Shushi Nishiwaki, Munekazu Tanaka, Reo Hata, Hirohiko Kohjitani, Koh Ono

**Affiliations:** Department of Cardiovascular Medicine, Graduate School of Medicine, Kyoto University, Kyoto, Japan

**Keywords:** Superior vena cava, Atrial tachycardia, Pulsed field ablation, Pulmonary vein isolation, Atrial fibrillation

**Key Teaching Points**

• Pulsed field ablation (PFA) delivered at the distal right superior pulmonary vein (RSPV), particularly with an olive-shaped application and close RSPV-superior vena cava (SVC) proximity on imaging, may be associated with unintended SVC electrical impact.
• When atrial tachycardia recurs after PFA-based pulmonary vein isolation, SVC upper loop macroreentry due to scar and/or slow-conduction zone in the SVC should be considered.
• Ablation strategy should be tailored to the mapped substrate: when the slow-conduction zone is confined to the SVC, the SVC isolation designed to include the slow-conduction zone may be effective.

## Introduction

Atrial fibrillation (AF) is the most common clinically significant cardiac arrhythmia. Catheter ablation is an established treatment for AF, with pulmonary vein isolation (PVI) as the cornerstone strategy.^1^^,^^2^ PVI used to be predominantly performed through conventional thermal methods that include radiofrequency, cryotherapy, and laser. However, pulsed field ablation (PFA) has been rapidly spreading because it causes less damage to adjacent tissues, such as the esophagus and the phrenic nerve, compared with thermal ablation.^3^ Furthermore, PFA generally does not cause pulmonary vein stenosis; hence, PFA application in a small olive-shaped configuration can be delivered within the pulmonary vein. This technique, known as olive strategy, has been reported to improve PVI durability.^4^ Although PFA has been widely regarded as a safe and promising modality, limited information exists regarding its potential effects on the superior vena cava (SVC), especially in relation to arrhythmogenesis. We report a rare case of the SVC reentrant atrial tachycardia (AT) that occurred shortly after PFA, highlighting a potential electrophysiological consequence of this novel technology.

## Case report

**Figure 1:**
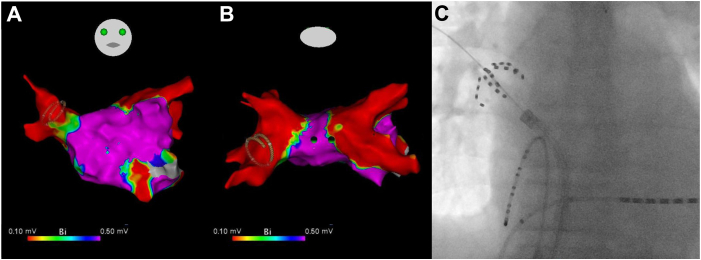
**A and B:** Voltage mapping after the initial PFA session with a pentaspline catheter at the RSPV showing complete pulmonary vein isolation. **C:** Fluoroscopic image of olive-shaped basket PFA application. PFA = pulsed field ablation; RSPV = right superior pulmonary vein.

A 57-year-old man presented with dyspnea on exertion and was diagnosed as having heart failure owing to persistent AF. He was referred to our institution for catheter ablation. Initially, echocardiography showed a reduced left ventricular ejection fraction of 30%, but it improved to 60% after sinus rhythm restoration with cardioversion. Coronary angiography revealed no significant stenosis, and endomyocardial biopsy showed no findings suspect to secondary myocarditis. Under deep sedation with dexmedetomidine and propofol (without muscle relaxant), PVI was performed using a pentaspline PFA catheter (FARAPULSE, Boston Scientific, Marlborough, MA) under electroanatomic mapping system guidance (CARTO, Biosense Webster, Irvine, CA). In addition to 2 basket and 2 flower applications to each pulmonary vein, additional applications were delivered to the antrum and the carina region. Furthermore, an additional application with olive-shaped configuration was delivered to the right superior pulmonary vein (RSPV), resulting in a total of 61 applications (Figure 1). Successful electrical isolation of all pulmonary veins was confirmed by postablation left atrial mapping. To prevent typical atrial flutter, empirical cavotricuspid isthmus (CTI) ablation was additionally performed using a radiofrequency ablation catheter (QDOT MICRO, Biosense Webster). Bidirectional block across the CTI was confirmed by differential pacing. Atrial arrhythmia induction test was not performed. No spontaneous AF or AT was observed after PVI and empirical CTI ablation, and the procedure was completed without complications.

**Figure 2:**
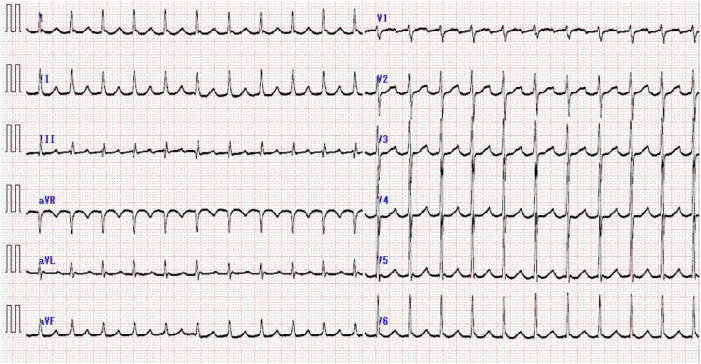
12-lead electrocardiogram of atrial tachycardia.

**Figure 3:**
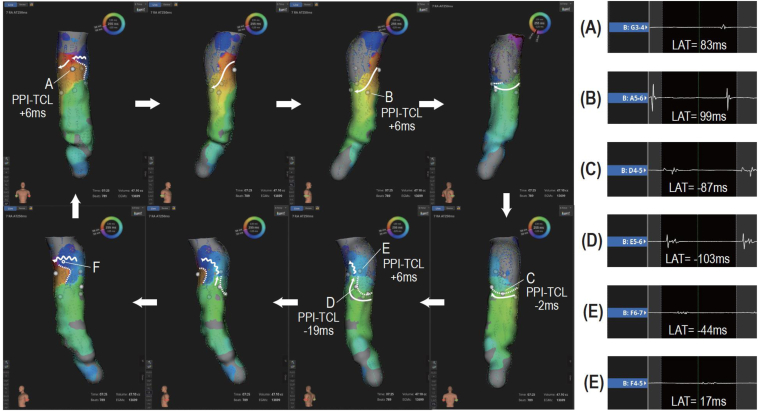
Activation map during AT demonstrating a reentrant circuit around a focal scar at the posterior SVC and slow conduction in the anterior-septal SVC. The *white solid arrows* represent the endocardial activation propagation (*straight arrows* indicate relatively preserved conduction, whereas *wavy arrows* indicate slow conduction). The *dashed lines* indicate the presumed lines of conduction block. The *white tags* (**A–E**) mark sites where entrainment pacing was performed. PPI-TCL values at entrainment sites are shown beneath each *white tag*. The *white circle* (**F**) indicates a site shown for an electrogram only. The panels on the right show the local electrograms and LAT at sites A–F. Double potentials at sites C and D support the presence of conduction block, whereas fractionated potentials at sites E and F are consistent with slow conduction in the anterior-septal SVC. AT = atrial tachycardia; LAT = local activation time; PPI = postpacing interval; SVC = superior vena cava; TCL = tachycardia cycle length.

**Figure 4:**
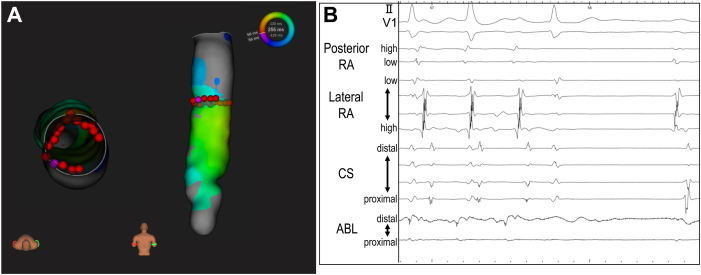
**A:** Radiofrequency ablation tags at the SVC. The *pink and red tags* indicate the AT termination site and endocardial radiofrequency ablation sites, respectively. **B:** Electrogram of AT termination during radiofrequency ablation at the septal aspect of the SVC. ABL = ablation catheter; AT = atrial tachycardia; CS = coronary sinus; RA = right atrium; SVC = superior vena cava.

The patient remained in sinus rhythm until discharge. However, 10 days after the initial procedure, the patient developed recurrent symptomatic AT documented on a 12-lead electrocardiogram (Figure 2). A second procedure was performed 2 months later. Voltage mapping using a multielectrode catheter (Orion, Boston Scientific) confirmed persistent isolation of all pulmonary veins and the CTI. Burst pacing induced the clinical AT with a tachycardia cycle length (TCL) of 250 ms, showing a concentric sequence in the coronary sinus. Activation mapping demonstrated a reentrant circuit around the posterior SVC scar and low-voltage area with slow conduction in the anterior-septal SVC, suggesting upper loop macroreentry (Figure 3, Supplemental Video). Representative electrograms along the anterior-septal SVC showed fractionated potentials consistent with slow conduction (Figure 3E and 3F). Entrainment pacing was performed at multiple sites around the SVC with a fixed cycle length (TCL −15 ms). The postpacing interval minus the TCL was less than 30 ms at all sites, confirming that the SVC was part of the reentrant circuit. Radiofrequency ablation was delivered along the SVC from the posterior aspect toward the anterior-septal aspect (25–30 W for 20–30 seconds per lesion). Before each application, high-output pacing from the ablation catheter (7V) was performed to assess phrenic nerve proximity, and radiofrequency ablation was avoided at sites where diaphragmatic capture occurred. When ablation reached the septal points, the AT terminated and turned to sinus rhythm (Figure 4B). Subsequently, circumferential ablation was performed to achieve the SVC isolation, including the anterior-septal slow-conduction zone identified on the activation map, while sparing the posterolateral SVC adjacent to the phrenic nerve (Figure 4A). The SVC isolation was confirmed by elimination of the SVC potentials and exit block with pacing from within the SVC. No AT was inducible with burst pacing, and the procedure was completed. No recurrence of AT or AF was observed during 3 months of follow-up.

## Discussion

**Figure 5:**
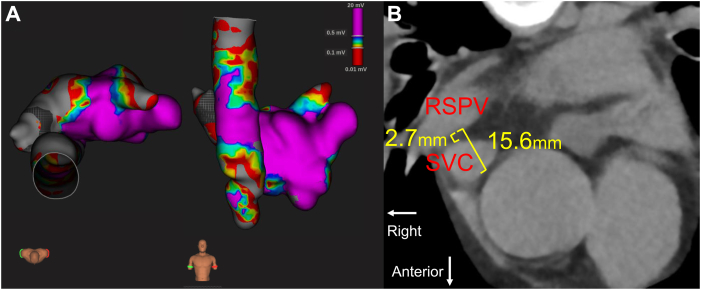
**A:** Voltage mapping of RA and LA during AT. The SVC is located in front of the upper branch of RSPV. A low-voltage area was identified in the anterior-septal SVC. **B:** CT findings. The distance between the RSPV and SVC was 2.7 mm posteriorly and 15.6 mm anteriorly. AT = atrial tachycardia; CT = computed tomography; LA = left atrium; RA = right atrium; RSPV = right superior pulmonary vein; SVC = superior vena cava.

SVC reentrant AT is a rare form of AT after AF ablation because a longitudinal scar extending from the SVC to the right atrium (RA) is the prerequisite substrate.^5^ Actually, 87.5% of patients with the SVC reentrant AT had a history of cardiac surgery.^6^ In our patient without cardiac surgery, the reentrant circuit was formed by a posterior scar and an anterior-septal slow-conduction zone. The RSPV and posterior aspect of the SVC are in close proximity; hence, ablation at the distal RSPV may affect the adjacent SVC. Thermal ablation near the RSPV has occasionally been associated with the SVC conduction delay or isolation in the adjacent SVC owing to this anatomic relationship.^7^ In particular, with cryoballoon ablation, a shorter RSPV-SVC distance correlates with greater electrophysiological effects.^8^ Recent reports have described frequent SVC electrical impact after PFA for the RSPV.^9^ Moreover, a pentaspline PFA catheter has been reported to cause the SVC impact more frequently than the variable-loop circular PFA catheter (88% vs 59%; *P* = .0009).^9^ Although SVC electrical impact after PFA has been reported, to the best of our knowledge, this is the first case report in which such SVC impact may have influenced the clinical course by leading to recurrent SVC upper loop macroreentry. In animal experiments, PFA produced dense transmural scarring extending approximately 10 mm.^10^ In our patient, the SVC was located in front of the upper branch of RSPV (Figure 5A), with the distance of 2.7 mm between the 2 veins (Figure 5B). In our procedure, the olive-shaped basket application at the distal RSPV may have directed toward the SVC. These findings suggest a possible association between a posterior SVC scar and PFA delivered at the distal RSPV. However, because RA and SVC voltage mapping was not performed before PFA at the initial procedure, the presence of a preexisting low-voltage substrate cannot be completely excluded. Accordingly, this case suggests that PFA near the RSPV may unintentionally affect the SVC, potentially creating a substrate for reentrant AT.

As factors to explain recurrent SVC upper loop reentrant AT, we considered not only a simple dense scar in the posterior SVC but also the anterior-septal slow-conduction zone. The distance between the RSPV and SVC was 15.6 mm anteriorly (Figure 5B), suggesting that the anterior-septal SVC would usually be less affected by PFA at the RSPV. However, a recent study reported new low-voltage zones in the SVC after PFA in 82% of patients, as well as the circumferential SVC impact in 14.7% of patients. The study also found that patients with the SVC deformity showed a higher incidence of the circumferential SVC impact.^9^ These data suggest that when the distance between the anterior and posterior wall of SVC is short, PFA at the RSPV may affect the anterior SVC. In this case, although the shape of SVC appeared to be roughly circular in preoperative computed tomography (Figure 5B), compression of the basket-shaped PFA catheter at the distal RSPV may have flattened the SVC, causing incomplete lesion in the anterior-septal SVC.

For treatment of SVC upper loop macroreentry, previous substrate-based strategies often target a critical isthmus by creating a linear lesion from the SVC-related scar to the inferior vena cava (IVC) or tricuspid annulus.^6^ In our case, the posterior SVC scar extended inferiorly toward the IVC, and a SVC-to-IVC linear lesion would have been a conventional approach. However, because an anterior-septal slow-conduction zone participating in the circuit was identified on activation mapping, we were concerned that the SVC-IVC line alone could leave this slow-conduction substrate intact and potentially allow recurrence of an AT limited to the anterior-septal SVC. Accordingly, we selected the SVC isolation including the anterior-septal slow-conduction zone. Notably, no low-voltage area was identified in the RA body, and no AT was inducible with burst pacing after the SVC isolation; thus, an additional SVC-IVC linear ablation was not performed.

Our case suggests that attention to the SVC may be warranted when performing PFA near the RSPV, particularly when an olive-shaped distal application is used and preprocedural imaging shows close RSPV-SVC proximity. Although acute post-PFA RA/SVC mapping may not reliably predict chronic substrate because of reversible stunning, identifying a low-voltage area and/or slow-conduction zone around the SVC immediately after PVI may be clinically informative. Such findings may raise suspicion for SVC upper loop macroreentry if AT recurs after PFA-based PVI. Moreover, because the SVC can serve as a source of AF triggers,^11^ additional SVC isolation during the same procedure might be considered in selected cases, depending on the extent and distribution of PFA-related SVC impact. These considerations remain speculative and should be interpreted with caution; future studies are needed to clarify mechanisms and guide prevention.

## Conclusion

SVC upper loop macroreentry may occur after pentaspline PFA-based PVI, especially when distal RSPV applications are delivered in close proximity to the SVC.

## Disclosures

The authors have no conflicts of interest to disclose.

## Appendix Supplementary Data

Supplementary Video 1

Video Description
