# Supplementary material for: A case of superior vena cava reentrant atrial tachycardia after pulmonary vein isolation with pentaspline pulsed field ablation
Source: HeartRhythm Case Rep. 2026 Mar 24;12(7):697–701. doi: 10.1016/j.hrcr.2026.03.016 (PMC13379345; doi:10.1016/j.hrcr.2026.03.016)
Supplement: Video Description [file mmc2.docx]

**Video legend**

**Propagation of atrial tachycardia on activation mapping.**
